# Supplementary material for: Potential of fecal microbiota for early-stage detection of colorectal cancer
Source: Mol Syst Biol. 2014 Nov 28;10(11):766. doi: 10.15252/msb.20145645 (PMC4299606; doi:10.15252/msb.20145645)
Supplement: Supplementary file 1 — Supplementary Information [file msb0010-0766-sd1.pdf]

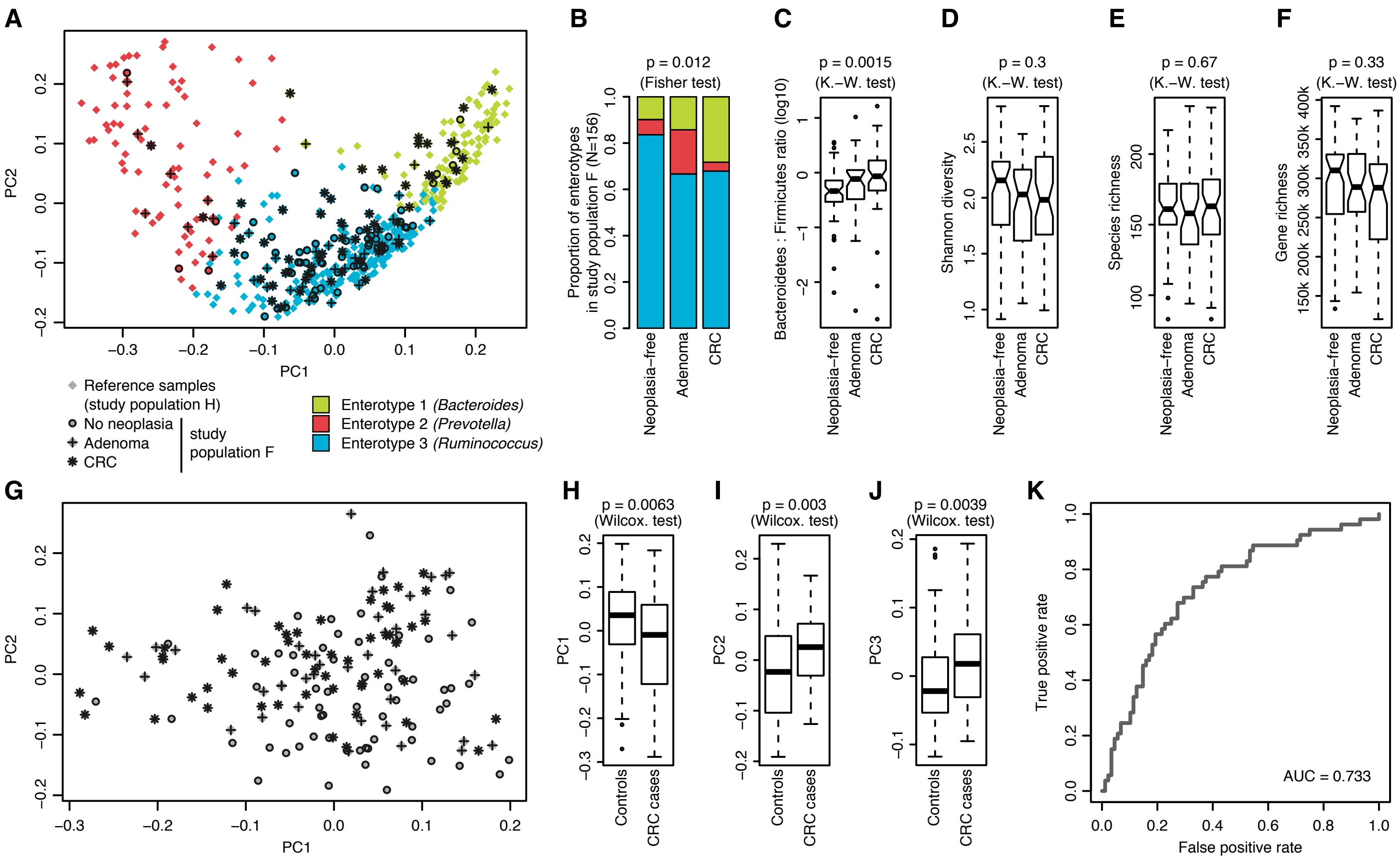

Supplementary Figure S1. Microbial community analysis of fecal samples from CRC patients, adenoma patients and neoplasia-free controls.

All boxplots show medians as horizontal thick lines within boxes that indicate the interquartile range (IQR). Whiskers extend up to the most extreme data point within 1.5 times the IQR. Outliers outside that range are drawn as circles.

(A) Enterotypes (Arumugam et al, 2011) of study population F in the context of all controls from study population H (see Methods and Arumugam et al, 2014 for details on enterotype assignments). Patient groups are indicated with different symbols (see key).

(B) Enterotype distribution in CRC patients and tumor-free controls (study population F).

(C) Abundance ratio between the Bacteroidetes and the Firmicutes phylum (Turnbaugh et al, 2006).

(D) Comparison of Shannon diversity (species level, see Methods for details) broken down by patient group (study population F).

(E) Comparison of observed species richness (that is the number of specI clusters, see Mende et al, 2013, with nonzero abundance) between patient groups (study population F).

(F) Comparison of gene richness patient groups (study population F, see Methods).

(G) Principal coordinate analysis of genus abundance profiles from participants of study population F. While conceptually similar to (A), this PCoA projection was done independently of any other data sets. Patient groups are the same as in (A).

(H-J) First three principal coordinate values plotted separately for CRC cases and a control group consisting of neoplasia-free participants and patients with small adenomas (large adenomas were excluded, see main text).

(K) Ten-fold cross-validation accuracy, evaluated by the receiver operating characteristic (ROC) curve, of a logistic regression model trained to distinguish CRC cases from the control group (using the same grouping as in (H)) based on the first ten principle coordinates (from (G)) and additionally the Bacteroidetes to Firmicutes abundance ratio from (C). Although CRC patients are significantly different from the control group in terms of principle coordinate (PC) projection (G-J) and differ significantly in terms of the Bacteroidetes to Firmicutes ratio (C), this model does not allow for accurate cancer detection (as compared to Fig 1, Supplementary Figs S3, S6 C-E and S10 C).

	CRC vs. Neoplasia-free		CRC vs. Adenoma		Adenoma vs. Neoplasia-free	
**Phyla (NCBI taxonomy)**						
Fusobacteria	↑	1.33E−05	↑	2.58E−04	—	
Firmicutes	↓	1.77E−03	—		—	
Actinobacteria	↓	4.58E−02	—		—	
Proteobacteria	↑	5.59E−02	↑	8.64E−02	—	
Bacteroides	↑	9.49E−02	—		—	
**Genera (NCBI taxonomy)**						
Fusobacterium	↑	2.72E−04	↑	2.06E−03	—	
Pseudoflavonifractor	↑	1.15E−03	—		—	
Eubacterium	↓	1.15E−03	↓	2.06E−03	—	
Ruminococcus	↓	6.87E−03	—		↓	6.73E−02
Peptostreptococcus	↑	1.29E−02	↑	9.01E−03	—	
Leptotrichia	↑	2.83E−02	—		—	
Porphyromonas	↑	5.59E−02	↑	9.01E−03	—	
Desulfovibrio	↑	5.59E−02	—		—	
Bifidobacterium	↓	5.59E−02	—		—	
Parvimonas	↑	5.77E−02	↑	1.90E−02	—	
Selenomonas	↑	6.43E−02	—		—	
Bilophila	↑	6.43E−02	—		—	
Campylobacter	↓	8.33E−02	—		—	
Acinetobacter	↓	8.33E−02	—		—	
Olsenella	—		↑	6.79E−02	—	
**Species (specI clusters)**						
Fusobacterium nucleatum subsp. *vincentii* [1482]	↑	1.30E−05	↑	5.12E−05	—	
Fusobacterium nucleatum subsp. *animalis* [1481]	↑	7.51E−05	↑	6.81E−04	—	
Fusobacterium nucleatum subsp. *nucleatum* [1479]	↑	6.54E−04	↑	2.32E−02	—	
Pseudoflavonifractor capillosus [1579]	↑	1.07E−03	—		—	
Fusobacterium nucleatum subsp. *polymorphum* [1480]	↑	3.23E−03	↑	7.12E−02	—	
Porphyromonas asaccharolytica [1056]	↑	9.61E−03	↑	6.46E−02	—	
unclassified *Ruminococcus* sp. [1621]	↓	1.73E−02	—		—	
unclassified butyrate−producing bacterium [1595]	↓	1.73E−02	—		—	
unclassified *Ruminococcaceae* bacterium [1580]	↑	1.73E−02	—		—	
Eubacterium hallii [1597]	↓	1.80E−02	—		—	
Eubacterium eligens [1627]	↓	2.05E−02	—		—	
Prevotella nigrescens [1069]	↑	2.15E−02	↑	5.99E−02	—	
unclassified *Ruminococcus* sp. [1620]	↓	2.20E−02	—		—	
Peptostreptococcus stomatis [1530]	↑	2.20E−02	↑	2.66E−03	—	
Leptotrichia hofstadii [1488]	↑	3.50E−02	—		—	
Streptococcus salivarius [1377]	↓	5.55E−02	—		—	
unclassified *Parvimonas* sp. [1506]	↑	5.63E−02	↑	9.43E−02	—	
Eubacterium rectale [1630]	↓	6.30E−02	↓	2.32E−02	—	
Fusobacterium periodonticum [1478]	↑	6.30E−02	—		—	
Roseburia intestinalis [1631]	↓	6.58E−02	—		—	
Parvimonas micra [1505]	↑	6.65E−02	↑	2.32E−02	—	
Bacteroides fragilis [1090]	↑	7.46E−02	—		—	
Eubacterium ventriosum [1629]	↓	7.70E−02	—		—	
Bilophila wadsworthia [756]	↑	8.41E−02	—		—	
unclassified *Neisseria* sp. [439]	↑	8.41E−02	—		—	
Campylobacter rectus [1720]	↑	8.41E−02	—		—	
Selenomonas sputigena [1654]	↑	8.41E−02	—		—	
Leptotrichia buccalis [1487]	↑	8.41E−02	—		—	
Clostridium hylemonae [1607]	↑	8.87E−02	—		—	
Ruminococcus bromii [1569]	↓	9.39E−02	—		—	
Clostridium symbiosum [1600]	↑	9.55E−02	—		—	
Olsenella uli [816]	—		↑	2.32E−02	—	
unclassified *Parvimonas* sp. [1507]	—		↑	2.32E−02	—	
Streptococcus anginosus [1394]	—		↑	6.29E−02	—	

Supplementary Figure S2. Microbial taxa with significantly different abundances in the three patient groups of study population F.

Significant differences in the relative abundance of phyla, genera and species (numbers in brackets indicating specI cluster identifiers from Mende et al, 2013) are shown for the three pair-wise comparison between the patient groups of CRC cases, participants with adenomas (of any size) and neoplasia-free participants. Significance was determined using FDR-corrected pair-wise Wilcoxon tests with a cutoff of 0.1 on the adjusted p-values (dashes indicate that a significant difference could not be detected at this cutoff). Red and green arrows denote the direction of change (abundance increase and decrease, respectively, in the first-mentioned group of the respective column header). The overlap and consistency in the differences between CRC versus neoplasia-free and CRC versus adenomas (first two columns) was tested for statistical significance using Fisher's exact test (on the 3 by 3 contingency table of increased, decreased and not significantly changed abundances) resulting in p-values of 0.11, 6.0E-06 and 2.6E-10 for the respective taxonomic ranks of phylum, genus and species. Except for the *Ruminococcus* genus, significant differences could not be detected between adenoma patients and neoplasia-free controls (last column).

We moreover assessed to which extent changes were robust to excluding patients with large adenomas (>10 mm in size) from the adenoma group. Arrows highlighted in shaded gray boxes indicate that these comparisons were also significant when large adenomas were excluded; the result is consistent with reduced statistical power in comparisons with an adenoma group of reduced size. The only additional significant changes seen in comparisons between CRC patients and patients with small adenomas (in contrast to all adenomas) were *Methanosphaera stadmanae* [94] and the corresponding genus *Methanosphaera* with decreased abundance in CRC.

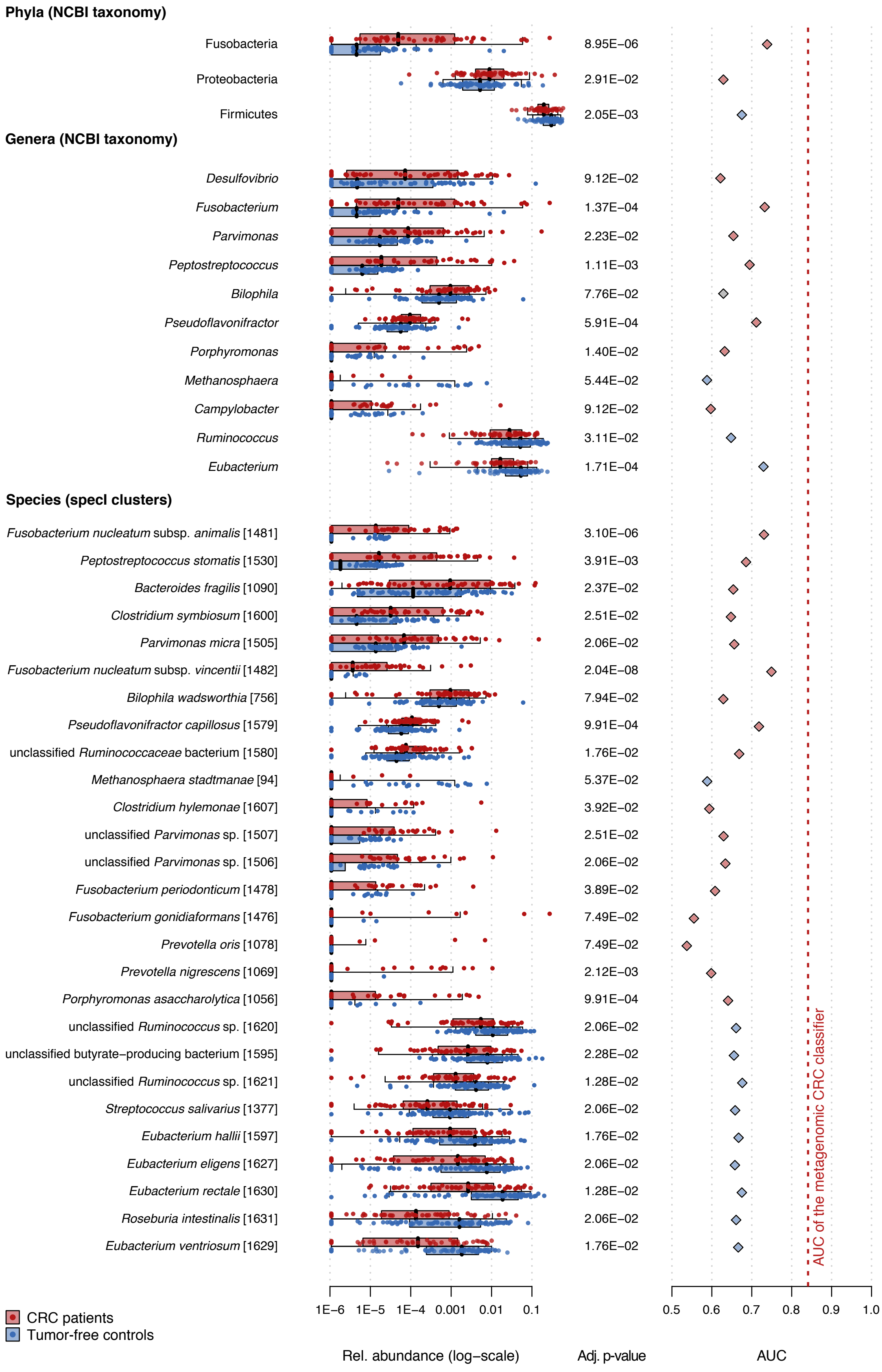

Supplementary Figure S3. Microbial taxa significantly associated with CRC in study population F. Differences in the relative abundance of phyla, genera and species (numbers in brackets indicating specI cluster identifiers from Mende et al, 2013) in a comparison of CRC patients to the control group, consisting of neoplasia-free participants and ones with small adenomas, (see key) were assessed using the Wilcoxon test. Shown are taxa with an FDR-corrected p-value < 0.1 (see Methods for details). The utility of each taxon as a potential CRC marker is assessed by the area under the ROC curve (AUC). As a ground truth for ROC analysis, colonoscopy outcomes were used (the dashed red vertical line indicates the accuracy of the metagenomic classifier for comparison, see Fig 1B).

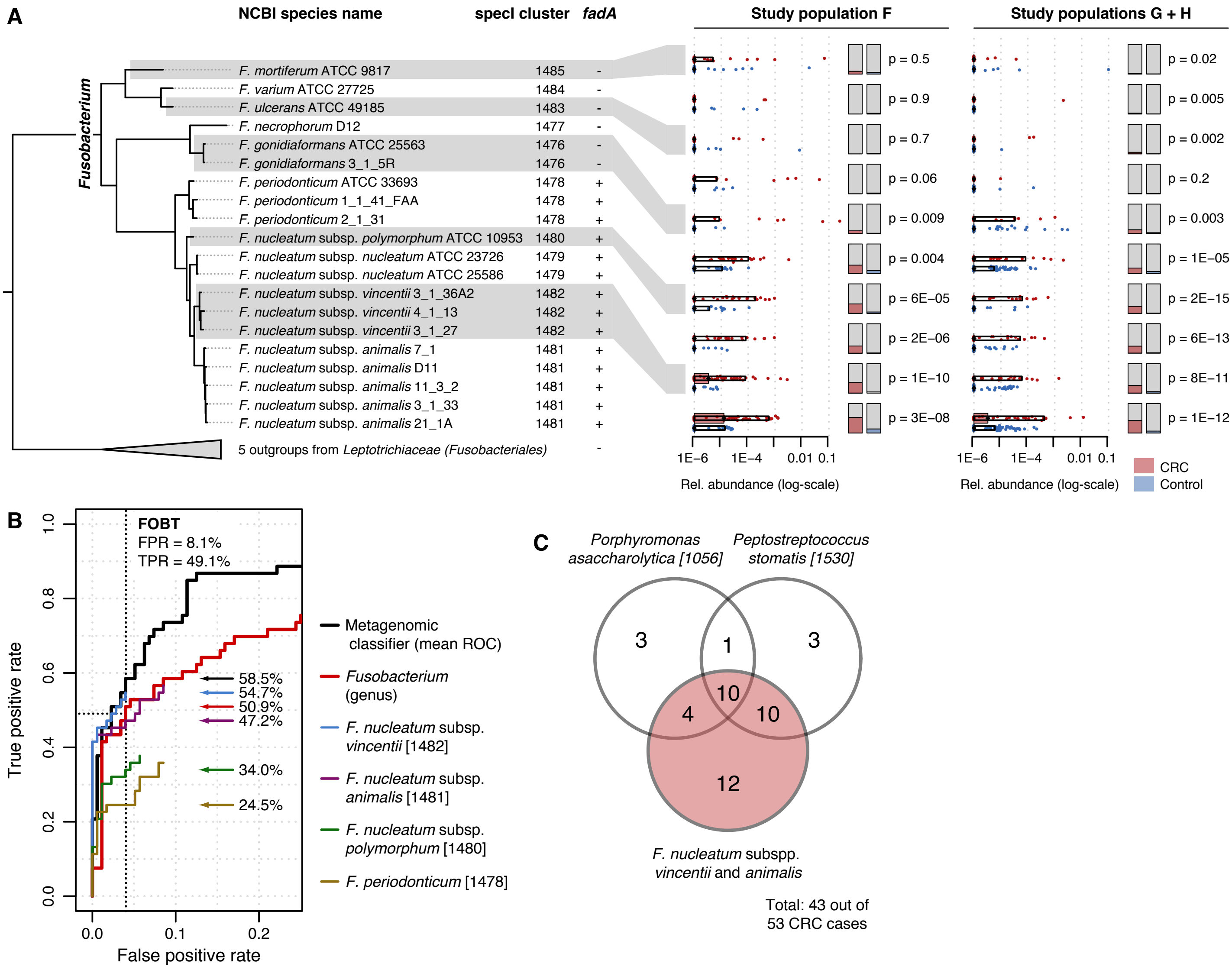

Supplementary Figure S4. Performance comparison of the metagenomic CRC classifier to individual markers including *Fusobacterium* species.

(A) *Fusobacterium* species and their abundance and prevalence in CRC. Species clusters generated with specI (Mende et al, 2013) are consistent with a marker-gene based maximum likelihood phylogeny (dendrogram, see Mende et al, 2013) and support the view that *Fusobacterium nucleatum* subspecies qualify as independent species. The presence of the *F. nucleatum fadA* gene, recently shown to be required for adherence, virulence, and tumorigenesis (Rubinstein et al, 2013), is indicated for each species cluster. Relative abundance and prevalence of Fusobacterium species in fecal CRC microbiomes relative to controls (participants with small adenomas or without any neoplasia) are plotted as colored dots; black boxes denote the interval between the 10th and 90th percentile of relative abundance with colored horizontal bars extending to the median, vertical bars display the prevalence (prev.). Graphs show that differences in prevalence between cases and controls are strongest for *F. nucleatum* subspp. *vincentii* and *animalis* in both study populations. The nominal p-values shown result from unpaired Wilcoxon tests of comparing relative abundances between CRC patients and controls.

(B) Relative abundance of *Fusobacterium* species and genus-level total relative abundance as potential CRC markers were assessed as individual predictors of CRC using ROC analysis in comparison to the full LASSO model of the metagenomic classifier (for which the mean ROC curve is shown, see Fig 1 A and B and Methods). All *Fusobacterium* specI clusters (as shown in (A), cluster numbers in brackets, Mende et al, 2013) were tested, but only the four best-performing markers are shown for clarity (see legend). Arrows indicate true positive rates (TPR, sensitivity) of individual markers at the false positive rate (FPR) of the FOBT (dotted lines).

(C) Taking an FPR cutoff of 8.1% (as observed for the FOBT) for each individual maker species, we assessed how many of the 53 CRC patients in study population F could at best be detected by each of them. In this analysis we included the four most discriminative marker species (Fig 1): *Porphyromonas asaccharolytica*, *Peptostreptococcus stomatis*, and the *Fusobacterium* subspp. *vincentii* and *animalis*, which were summarized (using an or-combination of their predictions). Despite substantial overlap between the predictions of the novel CRC markers *P. asaccharolytica* and *P. stomatis* with the *Fusobacterium* markers, which were previously associated with CRC (Kostic et al, 2013; Rubinstein et al, 2013), seven cancer cases were not detectable with the latter alone; and when combined, *P. asaccharolytica* and *P. stomatis* showed a detection rate comparable to *Fusobacterium* markers (31 and 36 CRC cases detected respectively). Note however that this analysis, in contrast to the LASSO metagenomic classifier, is not guaranteed to maintain a reasonable overall FPR.

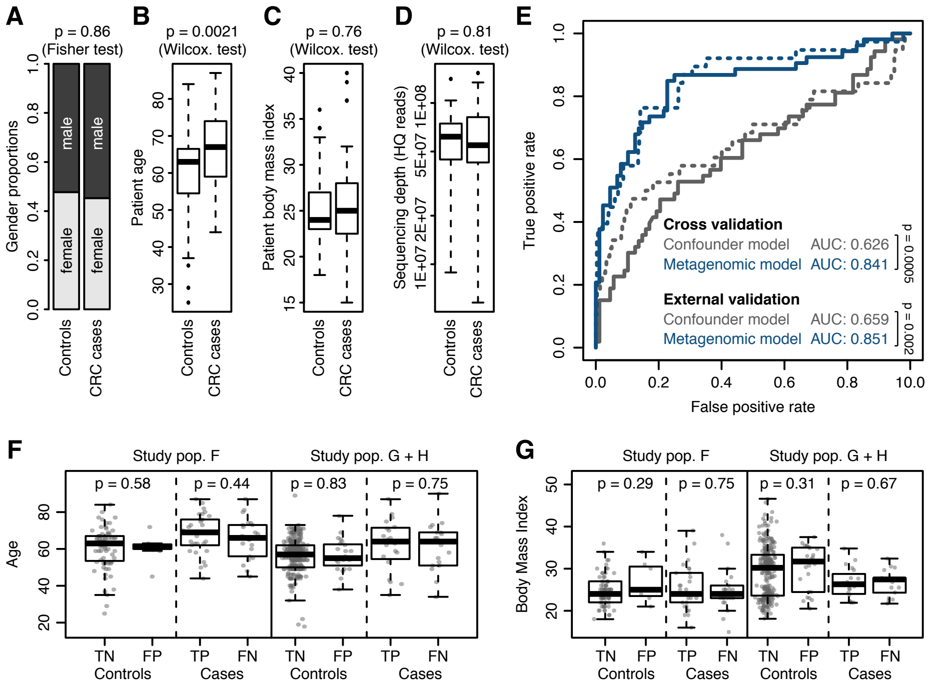

Supplementary Figure S5. Analysis of potential confounding factors that might affect the metagenomic CRC classifier.

All boxplots show medians as horizontal thick lines within boxes that indicate the interquartile range (IQR). Whiskers extend up to the most extreme data point within 1.5 times the IQR. Outliers outside that range are drawn as circles.

(A) Comparison of gender proportions between CRC patients and controls (with small adenomas or without any colonic neoplasia) of study population F.

(B) Comparison of patient age as a potential confounder (see main text and panels (E) and (F)).

(C) Comparison of body mass index (BMI) as a potential confounder (see main text and panels (E) and (G)).

(D) Comparison of sequencing depth between CRC patients and controls of study population F. Shown is the number of high-quality reads (used for abundance estimation, see Methods) on a log-scale.

(E) Accuracy (area under the ROC curve, AUC) of a logistic regression model trained to distinguish CRC cases from controls based on patient gender, age and BMI. Despite a significant age difference between CRC patients and controls (B), this model only achieves substantially (and significantly) lower accuracy as the metagenomic model both in ten-fold cross validation on study population F and in external validation on study populations G and H (see also Supplementary Figs S3, S6 C-E and S10 C).

(F) Metagenomic CRC predictions are unbiased for patient age, despite an age bias between cases and controls in the training set (B). The classifier neither shows a significant enrichment of old subjects among its false positive (FP) relative to its true negative (TN) predictions, nor a significant enrichment of young subjects among its false negative (FN) relative to true positive (TP) predictions. This observation is consistent between study population F used for cross validation and study populations G and H used for external validation.

(G) Metagenomic CRC predictions are unbiased for patient BMI. Details are as in (F).

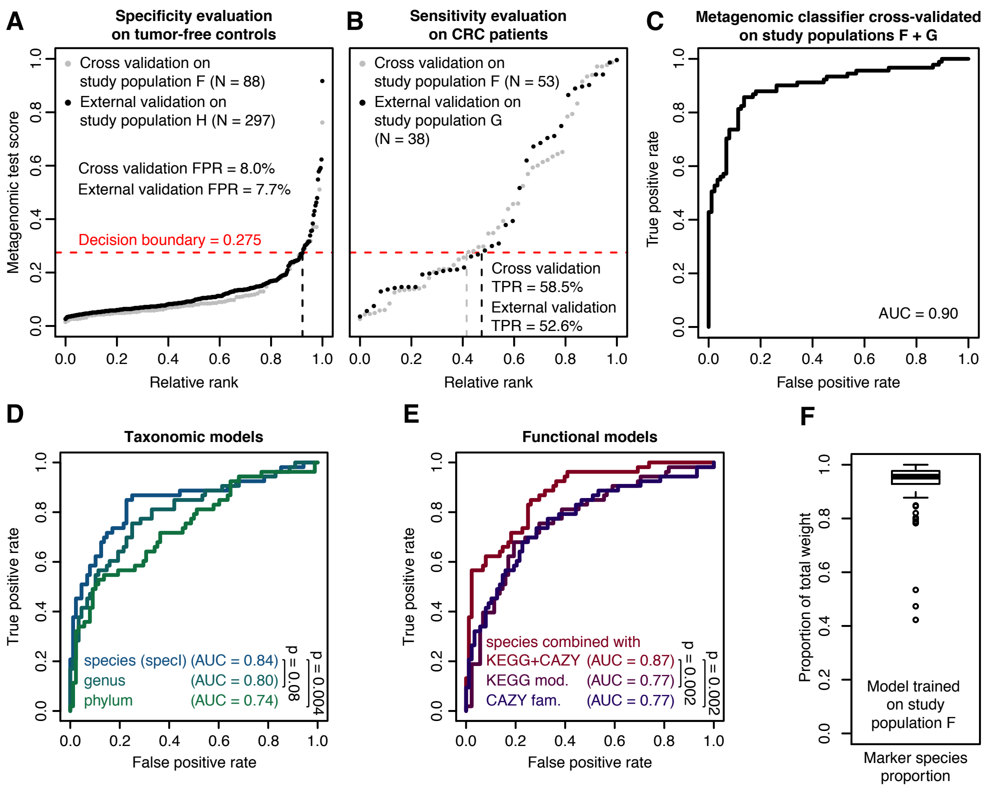

G

**Model trained on study population**<br>**Features utilized**	**F**<br>**species**		**F**<br>**spec. + FOBT**	**F + G**<br>**spec.**	**F**<br>**spec. + funct.**
**CRC marker species**	**Jackknife support**	**Model contribution**	**Model contribution**	**Model contribution**	**Model contribution**
Fusobacterium nucleatum subsp. *vincentii* [1482]	100%	23.4%	22.2%	19.4%	19.3%
Fusobacterium nucleatum subsp. *animalis* [1481]	100%	12.4%	8.6%	8.7%	10.3%
Peptostreptococcus stomatis [1530]	100%	9.4%	7.3%	4.4%	7.0%
Porphyromonas asaccharolytica [1056]	97%	6.4%	5.2%	13.0%	4.7%
Clostridium symbiosum [1600]	100%	5.7%	4.6%	9.0%	2.4%
Clostridium hylemonae [1607]	78%	2.3%	4.3%	NA	1.2%
Bacteroides fragilis [1090]	65%	1.3%	2.2%	NA	NA
Lactobacillus salivarius [1467]	55%	1.3%	NA	NA	NA
Fusobacterium gonidiaformans [1476]	56%	0.8%	NA	NA	NA
Lactobacillus ruminis [1466]	52%	0.5%	NA	1.4%	1.7%
Eubacterium rectale [1630]	54%	0.8%	1.7%	NA	NA
Bacteroides caccae [1096]	58%	0.8%	NA	NA	NA
Eubacterium ventriosum [1629]	52%	0.9%	NA	2.3%	NA
Clostridium scindens [1606]	56%	1.3%	NA	NA	1.2%
Eubacterium eligens [1627]	76%	1.5%	0.9%	NA	NA
Bifidobacterium angulatum [974]	72%	1.5%	NA	2.1%	0.8%
Methanosphaera stadtmanae [94]	79%	1.6%	3.1%	NA	1.9%
Dorea formicigenerans [1604]	72%	1.7%	2.5%	NA	NA
Butyrivibrio crossotus [1628]	82%	1.8%	0.9%	0.9%	0.9%
Phascolarctobacterium succinatutens [1659]	86%	2.9%	3.1%	5.0%	0.9%
unclassified *Ruminococcus* sp. [1620]	93%	5.5%	6.9%	3.8%	1.7%
Streptococcus salivarius [1377]	100%	9.2%	6.9%	10.8%	6.0%

Supplementary Figure S6. Additional information on the metagenomic CRC classifier.
(A) Specificity (1 – FPR) of the metagenomic test evaluated on study population H, which is not part of its cross-validation (training) set. The x-axis indicates the relative rank of the mean prediction score (across all classifiers from cross validation, see Methods) within study population H. In this graph, the FPR, defined as the number of false positive predictions (mean prediction score above the decision boundary of 0.275) among all controls, is indicated by the vertical dashed line (1 – its relative rank). For comparison, cross-validation results from study population F are also shown in gray. See also Fig 2.
(B) Sensitivity (TPR) of the metagenomic test (see Fig 1) evaluated on study population G, which is not part of its cross-validation (training) set. TPR is defined as the number of true positive predictions among all CRC patients for a decision boundary of 0.275 and denoted by the vertical dashed line (1 – its relative rank). Evaluation was relative to colonoscopy results as a ground truth. See Fig 2 and (A) for additional details.
(C) Cross-validation accuracy (ROC curve) of LASSO classifiers trained on species abundance profiles of samples from study populations F and G combined (N = 179) with the area under the curve (AUC) indicated (see Methods). Although it is difficult to rule out that due to the heterogeneity among CRC samples this classifier might also exploit confounding correlates, it illustrates the promise of larger study population for improved CRC detection accuracy.
(D) ROC curves for metagenomic CRC classifiers cross-validated on study population F with abundance profiles summarized at different taxonomic ranks as input features (see key and Methods). CRC detection accuracy deteriorates with lower taxonomic resolution at genus and phylum ranks compared to the classifier trained on species abundance profiles (shown in Fig 1, see also Supplementary Fig S3).
(E) ROC curves for metagenomic classifiers using functional abundance profiles summarized at the level of KEGG modules or CAZy gene families cross-validated on study population F (see key and Methods). Additionally a metagenomic classifier is included that is based on a combination (concatenation) of species abundance profiles, KEGG and CAZy abundance profiles achieving an AUC of 0.87, which is better than any taxonomic or functional model (see also panel (D) and Fig 1).
(F) Percentage of total weight attributed to the marker species as listed in the second column of panel (G). Features are only shown if they have a non-zero coefficient in at least 50% of the LASSO models from cross validation. Their relative weights is summed up in each model and summarized across all cross-validation models in the boxplot (see Methods and Supplementary Fig S1 for definition of boxplots).
(G) Additional information on markers from the metagenomic classifiers. First column: Jackknife support for each microbial marker, i.e. percentage of LASSO models (from cross validation) in which a feature corresponding to a microbial species has a non-zero coefficient; second column: percentage of total weight of each marker species in the model shown in Fig 1, A and B; third column: percentage of total weight of each marker species that is present in the model trained on metagenomic species abundance profiles and the FOBT test as an additional predictor; fourth column: percentage of total weight of each marker species that is present in the model cross-validated on study populations F and G (see panel (C)); fifth column: percentage of total weight of each marker species that is present in the model trained on species abundance and functional profiles, where the latter were a combination of KEGG module and CAZy family abundances (see panel (E)). NA represents features with a zero coefficient in at least 50% of the respective models (see main text and Methods for details).

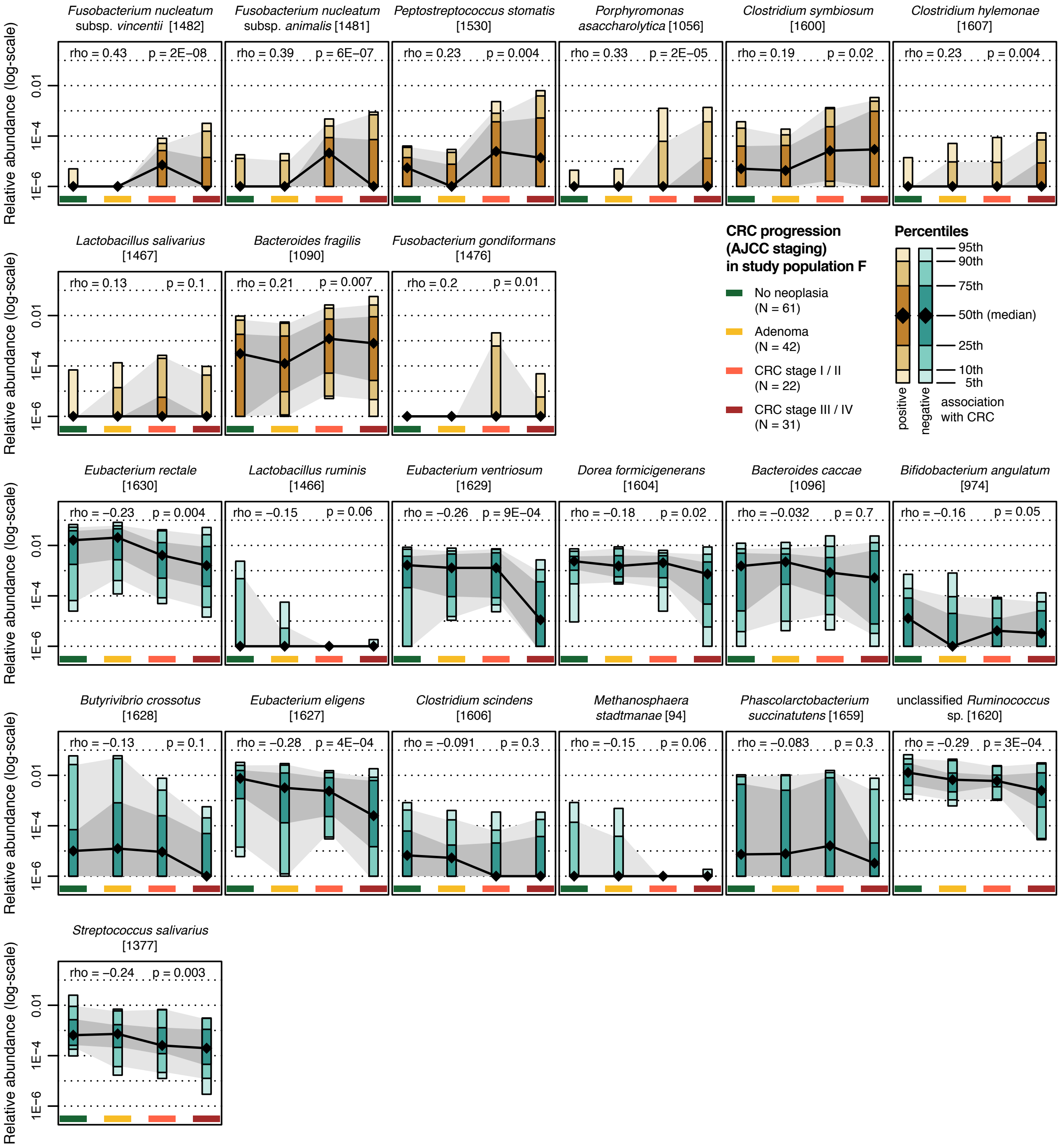

Supplementary Figure S7. Changes in relative abundance of the metagenomic marker species over the CRC progression from healthy participants over adenoma, early and late-stage cancer patients.
Relative abundance quantile ranges along CRC progression are shown as colored vertical boxes for each marker species and patient subgroup (same grouping as in Fig 1) with median values represented by black lines and diamonds (see legend). Patient subgroups are indicated by colored bars at bottom (see key, Table 1, Supplementary Table S1 and Supplementary Dataset S1). Spearman correlation strength (rho) between abundance changes of marker species (brackets indicate specI clusters, Mende et al, 2013) and progression, as well as its significance (FDR-corrected p-value) are shown at the top.

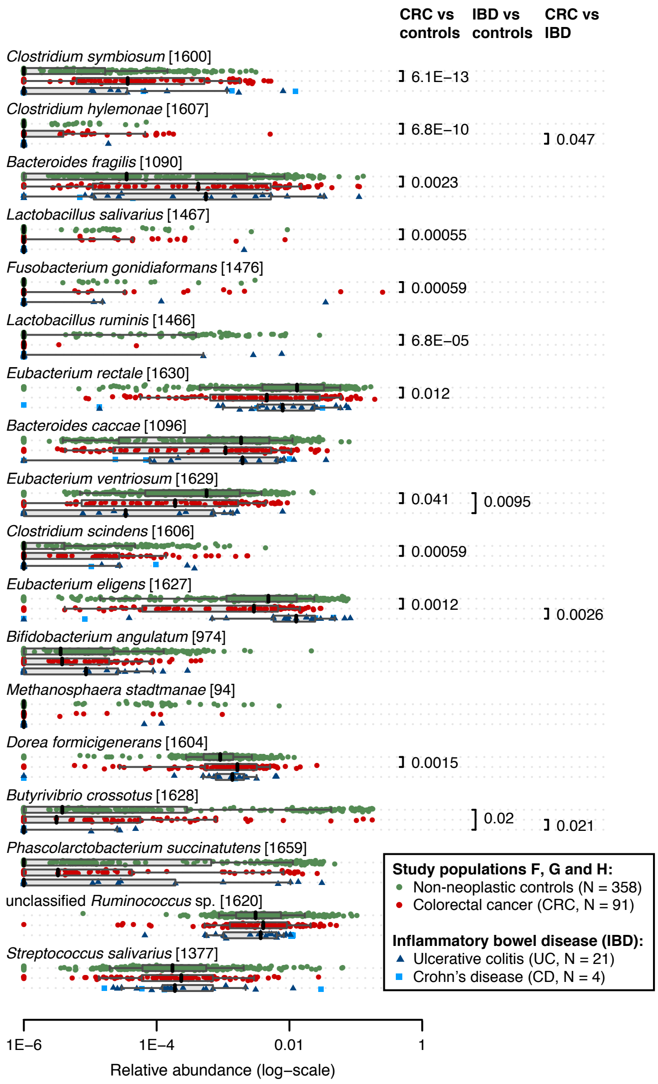

Supplementary Figure S8. Abundance of CRC marker species in IBD patients.
Comparison of the CRC microbial signature (see Fig 1A) to IBD microbiomes for the CRC marker species not shown in Fig 2B (see key, numbers in brackets indicate specI clusters, Mende et al, 2013; see Table 1, Supplementary Table S1 and Supplementary Dataset S1 for patient data). Abundance distributions are as in Fig 2B with significant differences between groups established by Wilcoxon test and FDR correction. Associations are generally stronger with CRC than with IBD with the exceptions of *Eubacterium ventriosum* and *Butyrivibrio crossotus*, both of which show a stronger decrease in IBD than in CRC.

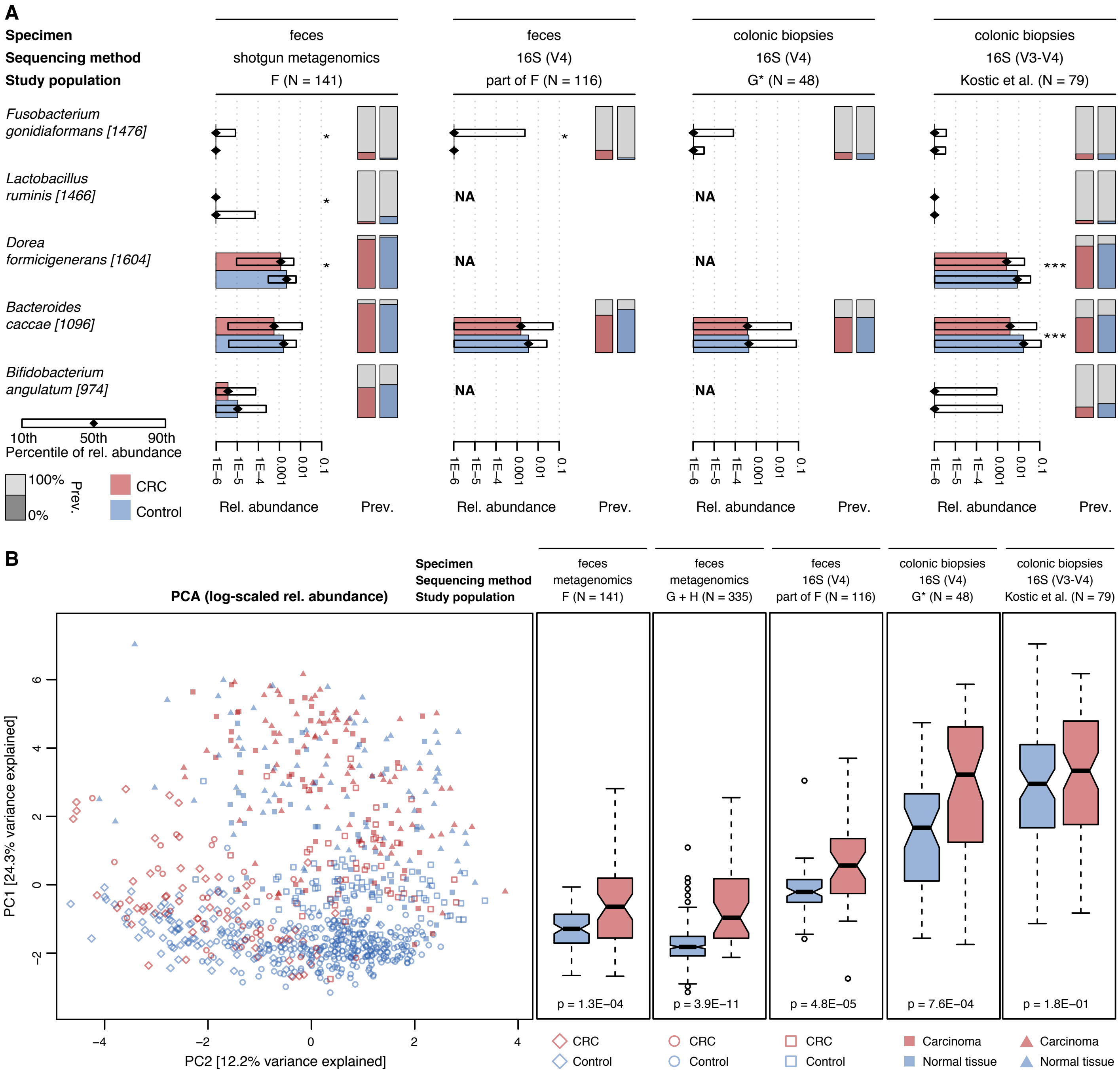

Supplementary Figure S9. Comparison of CRC-associated microbiota between tissue and fecal samples
(A) Consistency of CRC marker species abundances in fecal metagenomes and 16S rRNA profiles of tumor biopsies for markers not shown in Fig 3. Horizontal bars show CRC-associated changes in median relative (rel.) abundance of the marker species in the metagenomic CRC classifier. They are compared to 16S OTU abundances from a subset of fecal samples from study population F as well as two groups of patients in which microbial communities on tumor biopsies and healthy colonic mucosa were profiled and compared (of the 48 patients in study population G*, 13 are part of study population G; Kostic et al, 2012). Boxes denote the interval between the 10th and 90th percentile of relative abundance. Significance was assessed by unpaired and paired Wilcoxon tests for fecal and biopsy data sets, respectively. Vertical bars display the prevalence (prev.) of these marker species (percentage of individuals in which these species/OTUs had a rel. abundance exceeding 1E-05, see key).
A mapping between marker species and 16S OTUs could not be established for *Clostridium hylemonae*, *Lactobacillus salivarius*, *Butyrivibrio crossotus*, *Clostridium scindens*, *Methanosphaera stadtmanae* and *Phascolarctobacterium succinatutens* at 97% identity of the 16S rRNA fragment (see Methods for details on how marker species from metagenomics were mapped to 16S OTUs).
(B) Joint PCA of CRC tissue samples from this study and Kostic et al, 2012, and fecal samples from study population F (with taxonomic composition inferred by metagenomics and 16S amplicon sequencing, see key below boxplots) based on genera that are differentially abundant in at least one data set (see Methods for details). The first principal component (PC1), which accounts for ~24% of the total variance, shows a highly significant (Wilcoxon test) trend of separating CRC tissue/patients from normal tissue/tumor-free controls (see boxplots) that is shared between all data sets, despite the separation of fecal metagenomic samples from tissue 16S rRNA samples also being apparent in the PCA projection. Boxplots are as in Supplementary Fig S1.

A

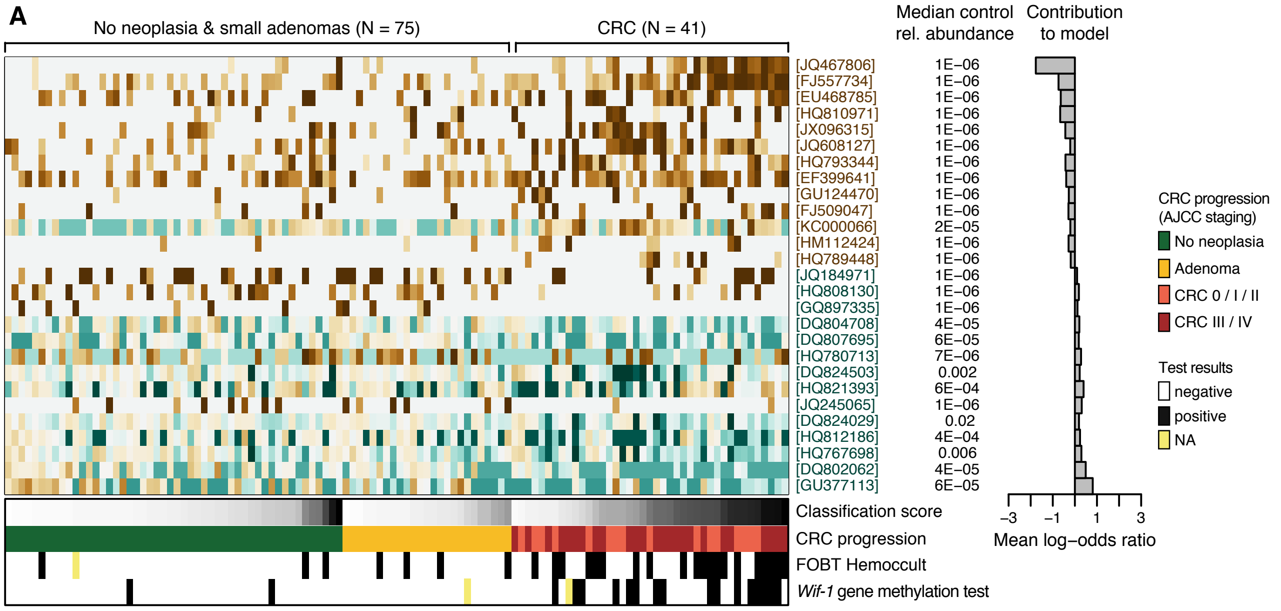

B

	Kingdom	Phylum	Class	Order	Family	Genus	Species
[JQ467806]	Bacteria	Fusobacteria	Fusobacteria	Fusobacteriales	—	—	—
[FJ557734]	Bacteria	Firmicutes	Clostridia	Clostridiales	Peptostreptococcaceae	*Peptostreptococcus*	—
[EU468785]	Bacteria	Firmicutes	Clostridia	Clostridiales	Ruminococcaceae	—	—
[HQ810971]	Bacteria	Firmicutes	Clostridia	Clostridiales	Clostridiaceae	*Clostridium*	—
[JX096315]	Bacteria	Firmicutes	Clostridia	Clostridiales	Lachnospiraceae	—	—
[JQ608127]	Bacteria	Firmicutes	Clostridia	Clostridiales	Lachnospiraceae	—	—
[HQ793344]	Bacteria	Bacteroidetes	Bacteroidia	Bacteroidales	Bacteroidaceae	*Bacteroides*	*Bacteroides oleiciplenus*
[EF399641]	Bacteria	Firmicutes	Clostridia	Clostridiales	—	—	—
[GU124470]	Bacteria	Firmicutes	Clostridia	Clostridiales	Eubacteriaceae	*Eubacterium*	—
[FJ509047]	Bacteria	Bacteroidetes	Bacteroidia	Bacteroidales	—	—	—
[KC000066]	Bacteria	Firmicutes	Clostridia	Clostridiales	Lachnospiraceae	—	—
[HM112424]	Bacteria	Proteobacteria	Gammaproteobacteria	Enterobacteriales	Enterobacteriaceae	—	—
[HQ789448]	Bacteria	Firmicutes	Clostridia	Clostridiales	Christensenellaceae	—	—
[JQ184971]	Bacteria	Firmicutes	Clostridia	Clostridiales	Lachnospiraceae	*Blautia*	—
[HQ808130]	Bacteria	Firmicutes	Clostridia	Clostridiales	Lachnospiraceae	—	—
[GQ897335]	Bacteria	Firmicutes	Clostridia	Clostridiales	Ruminococcaceae	—	—
[DQ804708]	Bacteria	Firmicutes	Clostridia	Clostridiales	Lachnospiraceae	*Pseudobutyrivibrio*	—
[DQ807695]	Bacteria	Firmicutes	Clostridia	Clostridiales	Lachnospiraceae	*Pseudobutyrivibrio*	—
[HQ780713]	Bacteria	Firmicutes	Clostridia	Clostridiales	Ruminococcaceae	—	—
[DQ824503]	Bacteria	Firmicutes	Clostridia	Clostridiales	Lachnospiraceae	*Dorea*	—
[HQ821393]	Bacteria	Bacteroidetes	Bacteroidia	Bacteroidales	Bacteroidaceae	*Bacteroides*	*Bacteroides caccae*
[JQ245065]	Archaea	Euryarchaeota	Methanobacteria	Methanobacteriales	Methanobacteriaceae	*Methanosphaera*	—
[DQ824029]	Bacteria	Firmicutes	Clostridia	Clostridiales	Lachnospiraceae	*Blautia*	—
[HQ812186]	Bacteria	Firmicutes	Clostridia	Clostridiales	Lachnospiraceae	—	—
[HQ767698]	Bacteria	Firmicutes	Clostridia	Clostridiales	Lachnospiraceae	*Anaerostipes*	—
[DQ802062]	Bacteria	Firmicutes	Clostridia	Clostridiales	Lachnospiraceae	*Pseudobutyrivibrio*	—
[GU377113]	Bacteria	Proteobacteria	Alphaproteobacteria	Sphingomonadales	Sphingomonadaceae	*Sphingomonas*	*Sphingomonas dokdonensis*

C

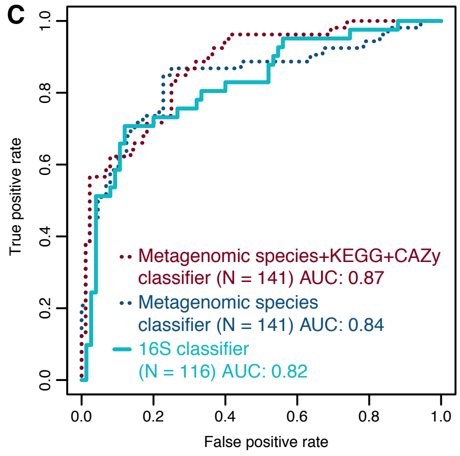

Supplementary Figure S10. A classifier based on 16S OTUs (clustered at 98% identity) from fecal samples can accurately detect CRC.
(A) Heatmap shows relative abundances of 16S OTUs that the classifier associated with CRC as fold change over the median relative abundance observed in controls (as indicated to the right). The mean contribution of each marker OTU to the classification is shown to the right with bar length corresponding to log-odds ratio in logistic regression (see Methods). Cancer stages are color-coded below the heatmap (see Table 1, Supplementary Table S1 and Supplementary Dataset S1 for patient data). Below, the mean test classification score from cross validation is shown as gray scale (using colonoscopy results as a ground truth). Displayed alongside are the results of the standard Hemoccult FOBT test and the *wif-1* gene methylation test (Lee et al, 2009; Mansour & Sobhani, 2009; see main text and Fig 1 for details).
(B) Consensus taxonomy of 16S OTUs from (A). Identifiers correspond to SILVA SSU Ref version 115 (Pruesse et al, 2007). Taxonomic annotations were generated by mapping all SILVA sequences to the NCBI taxonomy and determination of the lowest common ancestor of all taxonomically annotated sequences within each OTU cluster (see Methods). Dashes indicate that at this (and lower) taxonomic ranks annotations were either not available or inconsistent.
(C) ROC curves comparing the accuracy of the 16S classifier to the metagenomic classifiers that are either based on species abundance profiles (Fig 1 and Supplementary Fig S6 D) or on a combination of species profiles and functional abundance profiles (that is a concatenation with KEGG module and CAZy gene family abundances, see Supplementary Fig S6 E).

1E!

Supplementary Table S1. Overview of minimal metadata of study population F, G and H. Data are summarized by median with the interquartile range in brackets, n.a.: data not available.

Population	Disease status	Gender (M / F)	Age (years)	BMI (kg / m²)	Localization				
					RC[a]	RC/LC[b]	LC[c]	Sigma	Rectum
**F (N=156)**	Healthy	28/33	63.0 (56.0-67.0)	24.0 (23.0-26.5) (2 n.a.)	-	-	-	-	-
	Small adenoma	18/9	62.0 (53.0-66.0)	25.0 (23.0-29.8) (1 n.a.)	8	2	6	5	6
	Large adenoma	12/3	68.0 (62.5-71.0)	26.0 (23.0-27.5)	4	4	6	0	1
	AJCC stages I, II	10/12	70.5 (62.3-75.5)	26.0 (24.0-30.0) (1 n.a.)	6	0	8	3	5
	AJCC stages III, IV	19/12	65.0 (58.5-73.5)	24.0 (22.0-26.0) (1 n.a.)	11	0	15	1	4
**G (N=38)**	AJCC stages 0, I, II	13/12	65.0 (55.0-70.0)	27.0 (25.0-30.0)	7	0	2	7	9
	AJCC stages III, IV	12/1	63.0 (51.0-74.0)	26.0 (23.0-28.0)	4	0	1	4	4
**H (N=297)**	Healthy	130/162 (1 n.a.)	56.0 (50.0-61.0) (1. n.a.)	30.6 (23.7-33.6) (1. n.a.)	-	-	-	-	-

[a]RC: Right colon
[b]RC/LC: Multiple events localized right and left.
[c]LC: Left colon

Supplementary Table S2. Genes encoding bacterial toxins with potentially carcinogenic properties in fecal readouts.

For several bacterial genotoxins (e.g. *B. fragilis* toxins (BFTs) or Colibactin produced by some *E. coli* strains) and related gene families, which e.g. encode bacterial secretion systems, a role in the etiology of gastrointestinal diseases including colorectal cancer has been discussed. To be able to more comprehensively explore these, we performed targeted functional analyses (in addition to the unsupervised approach based on the KEGG and CAZy databases, which only provides limited coverage of these microbial functions, see Methods). We analyzed 15 specific bacterial toxin families and virulence factors discussed in the context of gastrointestinal disorders (see Dutilh et al, 2013; Fasano, 2002; Rubinstein et al, 2013). Out of these, we only found the *fadA* adhesin gene of *F. nucleatum* to be significantly enriched in fecal metagenomes from CRC patients of study population F. We thus neither detected a general enrichment of bacterial toxins, previously discussed in the context of CRC, nor do our results strongly suggest a dominant role for any factor in addition to *FadA*, which was recently shown to be required for *Fusobacterium* adhesion, virulence and promotion of tumorigenesis (Kostic et al, 2013; Rubinstein et al, 2013).

Toxin/family	Tested members	NCBI accession numbers	Length (aa)	e-value cutoff[a]	p-value[b] Prevalence of genes in patients and controls	Association with GI disorders	References
**Colibactin**<br>**(polyketide synthase (pks) island)**<br>**(*Escherichia coli*)**	clbJ:<br>Putative non-ribosomal peptide synthetase	WP_001518704	2166	1E-05	P=0.164	pks island encodes the genotoxin colibactin reported to promote DNA damage in eukaryotic cells	(Arthur et al, 2012; Cuevas-Ramos et al, 2010; Dutilh et al, 2013; Nougayrede et al, 2006)
		YP_001452455	2166		1		
		YP_006635482	2113		1		
		YP_005081859	2200				
		WP_001618609	2154				
		YP_001452456	2177				
	clbB:<br>Putative hybrid polyketide non-ribosomal peptide synthase synthase	WP_001616856	3206	1E-05	P=0.019		
		YP_006106330	3206		1		
		WP_004148958	3208		1		
		WP_010329099	3032				
		YP_005081866	3234				
	Prophage Integrase	NP_754341	423	1E-13	P=0.112		
		YP_007386848	418		0.981		
		NP_669700	420		1		
		WP_000058783	420				
		YP_006635556	424				
		WP_004961205	422				
		WP_000055687	420				
		WP_001527179	420				
	Thioesterase	NP_754343	240	1E-08	P=0.190		
		WP_004623651	235		1		
		YP_005147766	235		1		
		WP_007786651	270				
		WP_003206839	231				
		WP_010503396	239				
		YP_005146573	234				
		WP_006675300	243				
	clbC:<br>Putative polyketide synthase	NP_754360	869	1E-05	P=0.005		
		YP_669878	866		1		
		WP_001491526	866		1		
		YP_005081865	838				
		WP_020234547	705				
	clbH:<br>Putative non-ribosomal peptide synthase	NP_754353	1603	1E-42	P=0.233		
		YP_669873	1598		1		
		YP_005081861	1898		1		
		WP_004148953	1598				
		AGH69808	1349				
		WP_017314620	2002				
	clbA:<br>Putative 4'-	WP_001217108	244	1E-05	P=0.001		

	phosphopantetheinyl transferase	NP_754363	244		1		
		YP_007386769	244		1		
		WP_001560576	244				
		WP_020238096	248				
		WP_020236885	171				
		WP_019656694	268				
		WP_018455684	243				
		WP_007067260	248				
		AFB69912	240				
		WP_010120921	271				
	Penicillinbinding Protein PBP	YP_006101336	501	1E-22	P=0.275		
		WP_001491568	501		1		
		WP_020232476	520		1		
		YP_005081849	496				
		WP_007131910	508				
		YP_002505315	514				
		WP_001041646	508				
		WP_016077892	482				
		YP_005571592	482				
		WP_000751389	482				
		WP_016124690	482				
		YP_003664976	482				
		WP_016093005	482				
	Amidase	NP_754349	495	1E-39	P=0.0001		
		YP_669869	487		1		
		WP_001491570	487		1		
		WP_016529806	350				
		YP_003810088	490				
		WP_003882313	485				
		YP_007932719	496				
		WP_004928480	489				
		AGP56649	464				
**Shigella enterotoxin** ***(Shigella flexerneri*****)**	ShET1 enterotoxin (*Shigella flexneri 5a*)	-	-	-	n.a.	Toxin activates enterocyte signaling pathways contributing to diarrhea	(Dutilh et al, 2013; Fasano, 2002)
	shET2 enterotoxin (*Shigella flexneri 5a*)	NP_085167	572	1E-07	P=0.057		
		WP_005041841	569		0.868		
		WP_005017090	489		0.757		
		WP_005144365	424				
		CAA90938	565				
		NP_085251	565				
		YP_001919259	565				
		WP_000274016	455				
		WP_002954902	420				
		WP_001121628	549				
		WP_001121623	549				
		WP_001428909	455				
**Shiga toxin 1** **(*Shigella dysenteriae)***	A subunit	NP_288673	315	No hits	n.d.	Shiga toxins and shiga-like toxins block protein synthesis and are linked to haemorrhagic colitis	(Dutilh et al, 2013; Fasano, 2002)
		ABR09990	298				
		WP_000699959	315				
		1R4Q_A	293				
		WP_000691355	315				
	B subunit	NP_288672	89	No hits	n.d.		
		1410186B	89				
		WP_000722253	89				
		BAB83019	89				
		BAC10992	89				
		WP_000756806	89				
		AAQ16202	72				

		CAA46768	87				
**Shiga toxin 2**<br>(***Escherichia coli***)	A subunit and B subunit	NP_049500<br>AAM70045<br>CAX45706<br>WP_001452006<br>CAX45712<br>ACF16300<br>CAC48396<br>NP_543077	319<br>319<br>319<br>313<br>319<br>300<br>319<br>319	No hits	n.d.	subAB and shiga toxin 2 expression damages the colonic epithelium, induces necrosis, mononuclear inflammatory infiltration and mucin depletion	(Dutilh et al, 2013; Fasano, 2002; Gerhardt et al, 2013)
**CNF1 Cytotoxic necrotising factor 1** (***Escherichia coli***)		AAA85196<br>WP_000528124<br>WP_001537377<br>WP_001566411<br>WP_001102790<br>WP_005306733	1014<br>1014<br>1014<br>1014<br>1014<br>1037	No hits	n.d.	CNF1 is associated with cell transformation and protection of epithelial cells from apoptosis	(Dutilh et al, 2013; Travaglione et al, 2008)
**Subtilase cytotoxin** (***Escherichia coli***)	Subunit A (subA)	ACV40234<br>AEU11071<br>AEU11064<br>WP_000912969<br>AEU11070<br>AEU11068	351<br>342<br>342<br>347<br>316<br>338	1E-07	P=0.271<br>0.981<br>1	subAB and shiga toxin 2 expression damages the colonic epithelium, induces necrosis, mononuclear inflammatory infiltration and mucin depletion	(Dutilh et al, 2013; Gerhardt et al, 2013)
	Subunit B (subB)	ACV40235<br>AFX83960<br>YP_308821<br>WP_016603896<br>WP_016585489<br>WP_016256874	141<br>140<br>141<br>136<br>106<br>102	No hits	n.d.		
**Heat-labile enterotoxins**<br>(***Escherichia coli***)	Subunit LT-A	CAA23532<br>YP_006131768<br>YP_001451390<br>ABV01320<br>WP_001763691<br>ACU00910<br>BAG66065	254<br>276<br>269<br>258<br>218<br>258<br>178	No hits	n.d.	Heat labile toxins activate enterocyte signaling pathways and are related to diarrhea	(Dutilh et al, 2013; Fasano, 2002; Horstman & Kuehn, 2000; Kesty et al, 2004)
	Subunit LT-B	P0CK94<br>ABV01319<br>ABV01323<br>YP_006131769<br>ACJ23372<br>AAQ92973	124<br>124<br>124<br>124<br>104<br>99	No hits	n.d.		
**Heat-stable enterotoxin**<br>(***Escherichia coli***)	astA/EAST1	AAA20885<br>AAD43571<br>ADI59685<br>AAD43577<br>BAI44132<br>AAT12441<br>AAD43579	38<br>38<br>38<br>38<br>30<br>37<br>38	No hits	n.d.	Heat stable toxins activate enterocyte signaling pathways and are related to diarrhea	(Dutilh et al, 2013; Fasano, 2002; Konno et al, 2012)
**Heat-stable enterotoxin**<br>(***Escherichia coli***)	STa	YP_003294006<br>AAA24653<br>WP_001372581<br>WP_000733530<br>YP_003717630<br>WP_001694678	72<br>72<br>68<br>72<br>72<br>72	No hits	n.d.		(Dutilh et al, 2013; Fasano, 2002; Ngendahayo Mukiza & Dubreuil, 2013)
	STb	YP_006131763<br>YP_006940194<br>CAD87835<br>WP_000739297	71<br>71<br>71<br>71	No hits	n.d.		
**Enterotoxin**<br>(***Clostridium***	CPE	ADG84499<br>2XH6_A	312<br>319	No hits	n.d.	CPE induces of symptoms of food borne intoxication	(Dutilh et al, 2013; Fasano, 2002; Popoff,

***perfringens*)**		ACI16479<br>CAA57443<br>BAK40995<br>CAA04327	319<br>319<br>316<br>319				1998)
**Ribotype 01 toxin (*Clostridium difficile)***	Toxin A (tcdA)<br><br><br>Toxin B (tcdB)	AGG91503<br>AGG91562<br>AFN52237<br>YP_003213641<br>WP_009895695<br>AGG91599<br>CAA80815<br>AGG91603<br>EPZ61073<br>ADH94630<br>ADH94631<br>ADH94635	2710<br>2710<br>2710<br>2710<br>2084<br>2366<br>2367<br>2366<br>2364<br>2329<br>2328<br>2328	1E-78	P=0.015<br>1<br>1	Toxin A and B affect the intestinal permeability, cell adhesion and activation of apoptosis and causes diarrhea	(Dutilh et al, 2013; Fasano, 2002; Pothoulakis, 1996)
**C2 toxin (component 1) (*Clostridium botulinum*)**		CAA11969<br>2J3Z_A<br>BAA09942<br>YP_002650774<br>WP_019279183	431<br>431<br>431<br>431<br>431	0.0098	P=0.900<br>0.472<br>0.485	C2 toxin affects the enterocyte cytoskeleton by inactivation of Rho and actin	(Dutilh et al, 2013; Fasano, 2002)
***Bacteroides fragilis* toxin (BFT)**		BAA77276<br>WP_005800300<br>WP_005797262<br>3P24_A<br>BAA77277<br>AAB50410<br>BAA77275	397<br>405<br>405<br>397<br>397<br>389<br>397	No hits	n.d.	BFT triggers DNA damaging, colitis, cellular proliferation and colonic tumors	(Goodwin et al, 2011; Toprak et al, 2006; Wu et al, 2009)
**Enterotoxin *STN* (*Salmonella enterica*)**		AFN66163<br>AFN66161<br>AAA21354<br>AFN66162<br>AGR88902	195<br>194<br>249<br>194<br>249	No hits	n.d.	STN activates enterocyte pathways and is related to diarrhea	(Chopra et al, 1999; Fasano, 2002)
**Adhesion protein FadA** (***Fusobacterium nucleatum***)		AAW33965<br>WP_005895807<br>3ETZ_A<br>WP_009424473<br>WP_005967895<br>WP_008793520<br>YP_008019949<br>CDA08360<br>AAY47045<br>WP_008820435	129<br>129<br>119<br>128<br>128<br>133<br>129<br>129<br>129<br>128	1E-05	P=1.52E-07<br>0.321<br>0.029	*Fusobacterium nucleatum* carrying FadA adhesin promotes invasion and colorectal tumorigenesis and correlates with IBD.	(Rubinstein et al, 2013; Strauss et al, 2011)

[a]: e-value cutoffs for HMM prediction were set based on the optimization on an in-house protein catalogue collected

[b]: p-values were calculated using the Wilcoxon test

[c]: Logarithmic ratio of median abundance of patients to controls

n.d.: not detected in gene catalog

n.a.: no analysis possible

Supplementary References

Arthur JC, Perez-Chanona E, Muhlbauer M, Tomkovich S, Uronis JM, Fan TJ, Campbell BJ, Abujamel T, Dogan B, Rogers AB, Rhodes JM, Stintzi A, Simpson KW, Hansen JJ, Keku TO, Fodor AA, Jobin C (2012) Intestinal inflammation targets cancer-inducing activity of the microbiota. *Science* **338**: 120-123

Arumugam M, Raes J, Pelletier E, Le Paslier D, Yamada T, Mende DR, Fernandes GR, Tap J, Bruls T, Batto JM, Bertalan M, Borruel N, Casellas F, Fernandez L, Gautier L, Hansen T, Hattori M, Hayashi T, Kleerebezem M, Kurokawa K et al (2014) Addendum: Enterotypes of the human gut microbiome. *Nature* **506**: 516

Arumugam M, Raes J, Pelletier E, Le Paslier D, Yamada T, Mende DR, Fernandes GR, Tap J, Bruls T, Batto JM, Bertalan M, Borruel N, Casellas F, Fernandez L, Gautier L, Hansen T, Hattori M, Hayashi T, Kleerebezem M, Kurokawa K et al (2011) Enterotypes of the human gut microbiome. *Nature* **473**: 174-180

Chopra AK, Huang JH, Xu X, Burden K, Niesel DW, Rosenbaum MW, Popov VL, Peterson JW (1999) Role of Salmonella enterotoxin in overall virulence of the organism. *Microb Pathog* **27**: 155-171

Cuevas-Ramos G, Petit CR, Marcq I, Boury M, Oswald E, Nougayrede JP (2010) Escherichia coli induces DNA damage in vivo and triggers genomic instability in mammalian cells. *Proc Natl Acad Sci U S A* **107**: 11537-11542

Dutilh BE, Backus L, van Hijum SA, Tjalsma H (2013) Screening metatranscriptomes for toxin genes as functional drivers of human colorectal cancer. *Best Pract Res Clin Gastroenterol* **27**: 85-99

Fasano A (2002) Toxins and the gut: role in human disease. *Gut* **50**: III9-14

Gerhardt E, Masso M, Paton AW, Paton JC, Zotta E, Ibarra C (2013) Inhibition of water absorption and selective damage to human colonic mucosa are induced by subtilase cytotoxin produced by Escherichia coli O113:H21. *Infect Immun* **81**: 2931-2937

Goodwin AC, Destefano Shields CE, Wu S, Huso DL, Wu X, Murray-Stewart TR, Hacker-Prietz A, Rabizadeh S, Woster PM, Sears CL, Casero RA, Jr. (2011) Polyamine catabolism contributes to enterotoxigenic Bacteroides fragilis-induced colon tumorigenesis. *Proc Natl Acad Sci U S A* **108**: 15354-15359

Horstman AL, Kuehn MJ (2000) Enterotoxigenic Escherichia coli secretes active heat-labile enterotoxin via outer membrane vesicles. *J Biol Chem* **275**: 12489-12496

Kesty NC, Mason KM, Reedy M, Miller SE, Kuehn MJ (2004) Enterotoxigenic Escherichia coli vesicles target toxin delivery into mammalian cells. *EMBO J* **23**: 4538-4549

Konno T, Yatsuyanagi J, Saito S (2012) Virulence gene profiling of enteroaggregative Escherichia coli heat-stable enterotoxin 1-harboring E. coli (EAST1EC) derived from sporadic diarrheal patients. *FEMS Immunol Med Microbiol* **64**: 314-320

Kostic AD, Chun E, Robertson L, Glickman JN, Gallini CA, Michaud M, Clancy TE, Chung DC, Lochhead P, Hold GL, El-Omar EM, Brenner D, Fuchs CS, Meyerson M, Garrett WS (2013) Fusobacterium nucleatum potentiates intestinal tumorigenesis and modulates the tumor-immune microenvironment. *Cell Host Microbe* **14**: 207-215

Kostic AD, Gevers D, Pedamallu CS, Michaud M, Duke F, Earl AM, Ojesina AI, Jung J, Bass AJ, Tabernero J, Baselga J, Liu C, Shivdasani RA, Ogino S, Birren BW, Huttenhower C, Garrett WS, Meyerson M (2012) Genomic analysis identifies association of Fusobacterium with colorectal carcinoma. *Genome Res* **22**: 292-298

Lee BB, Lee EJ, Jung EH, Chun HK, Chang DK, Song SY, Park J, Kim DH (2009) Aberrant methylation of APC, MGMT, RASSF2A, and Wif-1 genes in plasma as a biomarker for early detection of colorectal cancer. *Clin Cancer Res* **15**: 6185-6191

Mansour H, Sobhani I (2009) Method, process, and kit for diagnosis or prognosis of colorectal cancer. WO 2009095596 A2. Paris, France.

Mende DR, Sunagawa S, Zeller G, Bork P (2013) Accurate and universal delineation of prokaryotic species. *Nat Methods* **10**: 881-884

Ngendahayo Mukiza C, Dubreuil JD (2013) Escherichia coli heat-stable toxin b impairs intestinal epithelial barrier function by altering tight junction proteins. *Infect Immun* **81**: 2819-2827

Nougayrede JP, Homburg S, Taieb F, Boury M, Brzuszkiewicz E, Gottschalk G, Buchrieser C, Hacker J, Dobrindt U, Oswald E (2006) Escherichia coli induces DNA double-strand breaks in eukaryotic cells. *Science* **313**: 848-851

Popoff MR (1998) Interactions between bacterial toxins and intestinal cells. *Toxicon* **36**: 665-685

Pothoulakis C (1996) Pathogenesis of Clostridium difficile-associated diarrhoea. *Eur J of Gastroenterol Hepatol* **8**: 1041-1047

Pruesse E, Quast C, Knittel K, Fuchs BM, Ludwig W, Peplies J, Glockner FO (2007) SILVA: a comprehensive online resource for quality checked and aligned ribosomal RNA sequence data compatible with ARB. *Nucleic Acids Res* **35**: 7188-7196

Rubinstein MR, Wang X, Liu W, Hao Y, Cai G, Han YW (2013) Fusobacterium nucleatum promotes colorectal carcinogenesis by modulating E-cadherin/beta-catenin signaling via its FadA adhesin. *Cell Host Microbe* **14**: 195-206

Strauss J, Kaplan GG, Beck PL, Rioux K, Panaccione R, Devinney R, Lynch T, Allen-Vercoe E (2011) Invasive potential of gut mucosa-derived Fusobacterium nucleatum positively correlates with IBD status of the host. *Inflamm Bowel Dis* **17**: 1971-1978

Toprak NU, Yagci A, Gulluoglu BM, Akin ML, Demirkalem P, Celenk T, Soyletir G (2006) A possible role of Bacteroides fragilis enterotoxin in the aetiology of colorectal cancer. *Clin Microbiol Infect* **12**: 782-786

Travaglione S, Fabbri A, Fiorentini C (2008) The Rho-activating CNF1 toxin from pathogenic E. coli: a risk factor for human cancer development? *Infect Agent Cancer* **3**: 4

Turnbaugh PJ, Ley RE, Mahowald MA, Magrini V, Mardis ER, Gordon JI (2006) An obesity-associated gut microbiome with increased capacity for energy harvest. *Nature* **444**: 1027-1031

Wu S, Rhee KJ, Albesiano E, Rabizadeh S, Wu X, Yen HR, Huso DL, Brancati FL, Wick E, McAllister F, Housseau F, Pardoll DM, Sears CL (2009) A human colonic commensal promotes colon tumorigenesis via activation of T helper type 17 T cell responses. *Nat Med* **15**: 1016-1022
